# Prognostic performance of multiple biomarkers in patients with acute coronary syndrome without standard cardiovascular risk factors

**DOI:** 10.3389/fcvm.2022.916085

**Published:** 2022-07-27

**Authors:** Le Wang, Hong-liang Cong, Jing-xia Zhang, Xi-ming Li, Yue-cheng Hu, Chen Wang, Jia-chun Lang, Bing-yang Zhou, Ting-ting Li, Chun-wei Liu, Hua Yang, Li-bin Ren, Wei Qi, Wen-yu Li

**Affiliations:** Department of Cardiology, Tianjin Chest Hospital, Tianjin, China

**Keywords:** prognosis, biomarker, acute coronary syndromes, standard cardiovascular risk factors, risk prediction

## Abstract

**Background and aims:**

Acute coronary syndrome (ACS) without standard modifiable cardiovascular risk factors (SMuRFs) represents a special case of ACS. Multiple biomarkers have been shown to improve risk stratification in patients with ACS. However, the utility of biomarkers for prognostic stratification in patients with ACS without SMuRFs remains uncertain. The aim of the present study was to evaluate the prognostic value of various biomarkers in patents with ACS without SMuRFs.

**Methods:**

Data of consecutive patients with ACS without SMuRFs who underwent coronary angiography in Tianjin Chest Hospital between January 2014 and December 2017 were retrospectively collected. The primary outcome was the occurrence of major adverse cardiovascular event (MACE), defined as a composite of cardiovascular death, myocardial infarction and stroke. Seven candidate biomarkers analyses were analyzed using models adjusted for established risk factors.

**Results:**

During a median 5-year follow-up, 81 of the 621 patients experienced a MACE. After adjustment for important covariates, elevated fibrinogen, D-dimer, N-terminal proB-type natriuretic peptide (NT-proBNP), and lipoprotein (a) [Lp(a)] were found to be individually associated with MACE. However, only D-dimer, NT-proBNP and Lp(a) significantly improved risk reclassification for MACE (all *P* < 0.05). The multimarker analysis showed that there was a clear increase in the risk of MACE with an increasing number of elevated biomarkers and a higher multimarker score. The adjusted hazard ratio- for MACE (95% confidential intervals) for patients with 4 elevated biomarkers was 6.008 (1.9650–18.367) relative to those without any elevated biomarker-. Adding- the 4 biomarkers or the multimarker score to the basic model significantly improved the C-statistic value, the net reclassification index and the integrated discrimination index (all *P* < 0.05).

**Conclusion:**

Fibrinogen, D-dimer, NT-proBNP and Lp(a) provided valuable prognostic information for MACE when applied to patients with ACS without SMuRFs. The multimarker strategy, which combined multiple biomarkers reflecting different pathophysiological process with traditional risk factors improved the cardiovascular risk stratification.

## Introduction

Although tremendous progress has been made in the evidence-based management of coronary artery disease (CAD), acute coronary syndrome (ACS) remains a major cause of mortality and morbidity worldwide (1). The key influences of diabetes mellitus, hypertension, hypercholesterolemia and smoking [known as the standard modifiable cardiovascular risk factors (SmuRFs)] on subsequent cardiovascular events has been well-illustrated (2). However, an increasing proportion of patients with ACS have no SmuRFs (3–9). Compared with patients with at least one risk factor, patients with ACS without SMuRFs remain at increased risk of death and recurrent cardiovascular events in contemporary secondary prevention strategies (5, 7). Therefore, it is important to further enhance risk stratification methods to identify patients with ACS without SMuRFs who have a higher risk for adverse events in so that appropriate treatment can be provided.

Several biomarkers play an important role in the risk stratification for recurrent cardiovascular risk in patients with ACS. Biomarkers representing the pathophysiological processes of hemodynamic stress [N-terminal pro-B-type natriuretic peptide (NT-proBNP)] (10), inflammation [high-sensitivity C-reactive protein (hs-CRP) and fibrinogen] (11, 12), activated coagulation (D-dimer) (13), endothelial activation [uric acid(UA)] (14), oxidative stress [gamma-glutamyl transferase (GGT)] (15), as well as lipid disorders[lipoprotein(a) [Lp(a)]] (16) have been shown to be associated with increased cardiovascular risk in patients with ACS. Moreover, a simultaneous multimarker strategy improved risk stratification compared to the use of individual biomarkers alone in patients with ACS (17–20). However, these biomarkers have not been individually or simultaneously evaluated in patients with ACS without SMuRFs.

It remains unclear whether these biomarkers are applicable to patients with ACS without SMuRFs for risk stratification in contemporary management. Therefore, the present study was conducted to investigate the prognostic performance of these established biomarkers and evaluate the effectiveness of a multimarker strategy in risk stratification in patients with ACS without SMuRFs.

## Methods

### Study population

**Figure 1:**
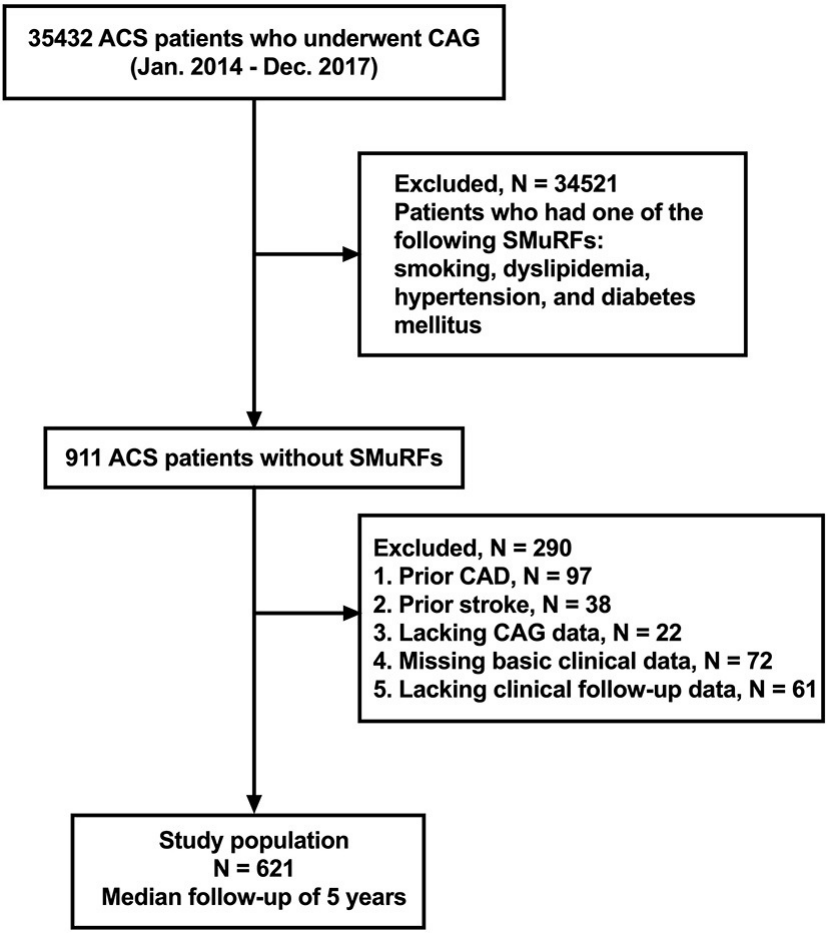
Flowchart of selection process and dropouts of the present study. ACS, acute coronary syndrome; CAG, coronary angiography; SmuRFs, standard modifiable cardiovascular risk factors; CAD, coronary artery disease.

This study was a retrospective, observational, single-center cohort study. From January 2014 to December 2017, 35,432 consecutive patients with ACS who underwent coronary angiography (CAG) in Tianjin Chest Hospital were enrolled in this study. The definition of ACS included either unstable angina pectoris (UAP), non-ST-segment elevation myocardial infarction (NSTEMI), and ST-segment elevation myocardial infarction (STEMI). A total of 34,521 patients who had one of the following SMuRFs were excluded: smoking, dyslipidemia, hypertension and diabetes mellitus. Patients meeting any of the following criteria were also excluded from the study: (1) patients with prior history of CAD (*n* = 97), (2) patients with history of stroke (*n* = 38), (3) patients lacking complete coronary artery angiography data (*n* = 22), (4) patients with missing data regarding baseline clinical data (*n* = 72), and (5) patients lacking complete clinical follow-up data (*n* = 61). Finally, a total of 621 patients with ACS without SMuRFs were included in this study (Figure 1). Patients' follow-up was conducted from December 2021 to January 2022 by telephone or *via* outpatient clinical visits. This study was approved by the local ethics committee and complied with the Declaration of Helsinki. Given the retrospective nature of this study, no written informed consent was obtained from-patients.

### Data collection and definitions

All data was collected from electronic medical records by 2 trained investigators who were blinded to the purpose of the study. The clinical data collected included age, gender, height, weight, heart rate (HR), systolic blood pressure (SBP), diastolic blood pressure (DBP), family history of coronary artery disease (CAD), clinical presentation, left ventricle ejection fraction (LVEF), the extent of the lesion including left main coronary artery disease and multi-vessel disease, revascularization, and medication at discharge. The following laboratory data were collected upon admission to hospital: creatine kinase (CK), creatine kinase-MB (CK-MB), high-sensitivity troponin T (hs-TnT), creatinine, fibrinogen, D-dimer, and N-terminal proB-type natriuretic peptide (NT-proBNP).Other laboratory data were collected under fasting conditions: fasting plasma glucose (FPG), total cholesterol (TC), triglycerides (TG), high-density lipoprotein cholesterol (HDL-C), low-density lipoprotein cholesterol (LDL-C), lipoprotein(a) [Lp(a)], gamma-glutamyl transferase (GGT), high-sensitivity C-reactive protein (hs-CRP), and uric acid (UA). All the laboratory measurements were conducted as part of routine medical practice. Plasma NT-proBNP levels were measured by electrochemiluminescence immunoassay method (Roche Diagnostics GmbH, Germany). Fibrinogen levels were measured by the Clauss method (Diagnostica Stago, France). Plasm hs-TnT was measured by using an electrochemiluminescence assay (Roche Diagnostics GmbH, Germany). Plasma D-dimer levels were measured by using immunoassay turbidimetry (Diagnostica Stago, France). The concentrations of CK, CK-MB and GGT were analyzed by rate method (Roche Diagnostics GmbH, Germany). Concentrations of FPG were measured by enzymatic hexokinase method (Roche Diagnostics GmbH, Germany). Levels of TG, TC, HDL-C, LDL-C and UA were measured by enzymatic colorimetry (Roche Diagnostics GmbH, Germany), whereas the concentration of hs-CRP was measured by immunoturbidimetry (Roche Diagnostics GmbH, Germany). Levels of Lp(a) were assessed using latex agglutination immunoassays (Roche Diagnostics GmbH, Germany) and presented in nmol/L. The method was not apo(a) isoform independent. Levels of LDL-C were corrected for Lp(a)- derived cholesterol which was calculated according to the formula [LDL-Ccorr = LDL–(Lp(a) × 0.3)].

Body mass index (BMI) was defined as weight (kg)/ [height (m)]^2^. Multivessel disease was defined as ≥ 2 vessels with ≥50% diameter stenosis as observed during CAG. The estimated glomerular filtration rate (eGFR) was calculated using the Modification of Diet in Renal Disease equation. Smoking included current or past smoking. Dyslipidemia was defined as TC ≥ 6.20 mmol/L or LDL-C ≥ 4.10 mmol/L or TG ≥ 1.70 mmol/L or HDL-C <1.04 mmol/L or having received treatment for dyslipidemia. Hypertension was defined as having been diagnosed with hypertension or having undergone previous antihypertensive therapy. Diabetes was defined as having been diagnosed with diabetes or having undergone previous glucose-lowering therapy. The Global Registry of Acute Coronary Events (GRACE) risk score was calculated according to eight variables on admission, including age, SBP, HR, presence of cardiac arrest during presentation, Killip class, ST-segment deviation, serum creatinine and positive cardiac biomarkers. The primary outcome was the occurrence of a major adverse cardiovascular event (MACE), defined as a composite of cardiovascular death, myocardial infarction and stroke.

### Statistical analysis

Continuous variables with normal distribution are presented as mean ± standard deviation, and-continuous variables with non-normal distribution are expressed as medians with interquartile ranges where a *t*-test or Mann-Whitney *U*-test was used. Categorical variables are presented as percentages and were compared using the chi-square test or Fisher's exact test. Natural logarithmic (log) transformation was performed for biomarkers with skewed distributions. A multivariate stepwise Cox regression analysis with entry/stay criteria of 0.1/0.1 was performed to determine the independent predictors of MACE. The possible independent predictors in the regression analysis did not include the biomarkers we studied herein. The possible factors (shown in Table 1) included LVEF, TG, eGFR, and hs-TnT. Finally, multivariate Cox regression analysis indicated that LVEF and eGFR independently predicted the occurrence of a MACE. Then, Cox proportional hazard regression models were used to evaluate the relationship between the levels of individual biomarkers we studied and MACE, and the hazard ratios (HRs) and 95% confidence intervals (CIs) were calculated. Model 1 was a univariate model of biomarkers. Model 2 was adjusted for LVEF and eGFR. Biomarkers with a statistically significant association with MACE were further analyzed. Each biomarker with a significant association was dichotomized according to the optimal cut-off values identified by receiver operating characteristic (ROC) curves. Multivariate Cox regression analysis was performed to evaluate the effect of multiple biomarkers on MACE as a categorical variable. Adjusted HRs and 95%CIs were calculated using the number of elevated significant biomarkers, taking the group without elevated biomarkers as reference. A multimarker risk score was calculated based on the significant biomarkers using the following equation: H = (β1x biomarker A) + (β2x biomarker B) + (β3x biomarker C) + (β4x biomarker D), where β denotes the estimate of beta coefficient for each biomarker, and A–D were obtained by fitting the Cox regression model for MACE (21). The patients were divided into quartiles according to their multimarker score. Adjusted HRs and 95% CIs were evaluated for each group, using the lowest quartile as reference. The cumulative incidence of MACE according to number of elevated biomarkers or the multimarker scores quartiles were estimated by using the Kaplan-Meier method, and compared by using the long-rank test. The C-statistics, net reclassification improvement (NRI), and integrated discrimination improvement (IDI) were calculated to assess the incremental predictive value of the biomarkers or the multimarker score over the basic model with employing only established risk factors. All two-sided *P*-values < 0.05 were considered statistically significant. All statistical analyses in this study were conducted using SPSS version 25.0 (IBM Corp, Armonk, New York) and SAS version 9.1.3 (Cary, NC, USA).

**Table 1:** Baseline characteristics of patients with and without MACE.

**Clinical characteristics**	**Total population**	**MACE**	**Non-MACE**	***P-*** **value**
	*N* = 621	*N* = 81	*N* = 540	
Age, years	63.9 ± 9.9	63.3 ± 10.8	64.0 ± 9.7	0.566
Male	321 (51.7)	43 (53.1)	278 (51.5)	0.788
BMI, kg/m2	24.2 ± 2.9	24.4 ± 3.3	24.2 ± 2.8	0.603
HR, bpm	69 (62–75)	70 (63–76)	69 (62–75)	0.772
SBP, mmHg	130 (120–138)	130 (120–138)	130 (120–138)	0.836
DBP, mmHg	75 (70–80)	75 (70–80)	75 (70–80)	0.585
Family history of CAD	81 (13.0)	14 (17.3)	67 (12.4)	0.224
Clinical presentation				0.265
UAP	450 (72.5)	54 (66.7)	396 (73.3)	
NSTEMI	62 (10.0)	12 (14.8)	50 (92.6)	
STEMI	109 (17.5)	15 (18.5)	94 (17.4)	
LVEF	60 (55–64)	59 (50–63)	60 (56–64)	0.035
GRACE Score	114 (99–133)	119 (99–139)	113 (99–133)	0.178
**Laboratory findings**				
FPG, mmol/L	5.2 ± 0.7	5.3 ± 0.9	5.2 ± 0.7	0.250
TC, mg/dl	175.3 ± 32.5	174.9 ± 32.9	175.3 ± 32.5	0.873
TG, mg/dl	105.4 (78.9–138.2)	97.5 (70.9–123.2)	105.4 (79.7–139.1)	0.060
HDL-C, mg/dl	49.1 ± 8.5	48.4 ± 8.5	49.5 ± 8.5	0.276
LDL-C, mg/dl	113.4 ± 29.4	112.6 ± 29.8	113.4 ± 29.4	0.848
Lp (a), nmol/L	38.4 (14.8–91.1)	52.8 (17.4–151.0)	36.3 (13.7–85.8)	0.011
GGT, IU/L	19 (14–29)	21 (14–29)	19 (14–30)	0.393
hs-CRP, mg/L	1.36 (0.61–3.82)	1.84 (0.77–6.41)	1.27 (0.58–3.58)	0.034
UA, umol/L	298.2 ± 77.0	302.8 ± 81.2	297.6 ± 76.4	0.568
Fibrinogen, g/L	3.39 ± 0.77	3.66 ± 0.98	3.35 ± 0.73	0.007
D-dimer, ug/ml	0.33 (0.24–0.50)	0.45 (0.28–1.32)	0.32 (0.23–0.46)	<0.001
eGFR, ml/min x 1.73 m^2^	87.6 ± 27.6	83.8 ± 26.7	91.6 ± 27.7	0.018
NT-proBNP, pg/ml	153.0 (63.3–582.5)	366.5 (128.8–890.7)	132.1 (59.1–495.5)	<0.001
CK, U/L	88 (66–139)	93 (69–275)	88 (66–135)	0.132
CK-MB, U/L	15 (12–24)	16 (12–37)	16 (12–24)	0.212
hs-TnT, pg/ml	0.010 (0.010–0.143)	0.020 (0.010–0.196)	0.010 (0.010–0.138)	0.064
Left main disease	59 (9.5)	10 (12.3)	49 (9.1)	0.349
Multi-vessel disease	417 (67.1)	59 (72.8)	358 (66.3)	0.242
Treatment				0.409
Medicine therapy	55 (8.9)	9 (11.1)	46 (8.5)	
PCI	515 (82.9)	63 (77.8)	452 (83.7)	
CABG	51 (8.2)	9 (11.1)	42 (7.8)	
Medications at discharge				
Aspirin	602 (96.9)	78 (96.3)	524 (97.0)	0.988
Clopidogrel/Ticagrelor	559 (90.0)	71 (87.7)	488 (90.4)	0.447
β-blocker	378 (60.9)	48 (59.3)	330 (61.1)	0.750
ACEI/ARB	137 (22.1)	20 (24.7)	117 (21.7)	0.540
Statin	591 (95.2)	75 (92.6)	516 (95.6)	0.378

*Data are expressed as mean ± SD, medians with interquartile ranges or percentage. BMI, body mass index; SBP, systolic blood pressure; DBP, diastolic blood pressure; HR, heart rate; CAD, coronary artery disease; UAP, unstable angina pectoris; NSTEMI, non-ST-segment elevation myocardial infarction; STEMI, ST-segment elevation myocardial infarction; LVEF, left ventricle ejection fraction; GRACE Score, Global Registry of Acute Coronary Events Score; FPG, fasting plasma glucose; TC, total cholesterol; TG, triglycerides; HDL-C, high-density lipoprotein cholesterol; LDL-C, low-density lipoprotein cholesterol; Lp(a), lipoprotein(a); GGT, gamma-glutamyl transferase; hs-CRP, high-sensitivity C-reactive protein; UA, uretic acid; eGFR, estimated glomerular filtration rate; NT-proBNP, N-terminal proB-type natriuretic peptide; CK, creatine kinase; CK-MB, creatine kinase-MB; hs-TnT, high-sensitivity troponin T;PCI, percutaneous coronary intervention; CABG, coronary artery bypass graft; ACEI, angiotensin II coenzyme inhibitor; ARB, angiotensin II receptor blocker*.

## Results

### Baseline characteristics

Of the 621 patients in the present study, 51.7% were men, and the mean age was 63.9 ± 9.9 years. Over an average of 5.0 years of follow-up, 81 patients (13.0%) experienced a MACE. The baseline characteristics of the patients are shown in Table 1. There were significant differences between the MACE group and the non-MACE group in LVEF (*P* = 0.035), Lp(a) (*P* = 0.011), hs-CRP (*P* = 0.034), fibrinogen (*P* = 0.008), D-dimer (*P* < 0.001), eGFR (*P* = 0.018), and NT-proBNP (*P* < 0.001). There were no significant differences between the two groups in other variables, including age, sex ratio, BMI, HR, SBP, DBP, family history of CAD, GRACE score, clinical presentation, FPG, TC, TG, HDL-C, LDL-C, non-HDL-C, TG/HDL-C, GGT, UA, CK, CK-MB, hs-TnT, left main coronary artery disease, multi-vessel disease, treatment, or medications at discharge.

### Single biomarker and clinical outcome

The biomarkers and risk of MACE are shown in Table 2. For ACS patients, the univariate cox regression analysis indicated that serum fibrinogen, log (hs-CRP), log (D-dimer), log (NT-proBNP) and log [Lp(a)] were associated with increased risk of MACE (all *P* < 0.05). After adjustments were made for LVEF and eGFR, the adjusted HRs of each 1-SD higher fibrinogen, log (D-dimer), log (NT-proBNP) and log [Lp(a)] were 1.200 (0.838–1.717), 6.028 (3.087–11.770), 2.000 (1.350–2.962), and 1.796 (1.149–2.806), respectively. For UAP patients, the univariate cox regression analysis indicated that serum fibrinogen, log (D-dimer) and log (NT-proBNP) were associated with increased risk of MACE (all *P* < 0.05). After adjustments were made for LVEF and eGFR, the adjusted HR of each 1-SD higher fibrinogen, log (D-dimer), and log (NT-proBNP) were 1.376 (1.022–1.851), 4.955 (2.051–11.971), and 4.073 (2.382–6.967), respectively. For NSTEMI patients, the univariate cox regression analysis showed that serum fibrinogen, log (D-dimer) and log [Lp(a)] were associated with increased risk of MACE (all *P* < 0.05). After adjustments were made for LVEF and eGFR, the adjusted HR of each 1-SD higher fibrinogen, log (D-dimer), and log [Lp(a)] were 3.684 (1.768–7.679), 21.265 (3.863–117.052), and 4.833 (1.108–21.069), respectively. For STEMI patients, the univariate cox regression analysis showed that log (D-dimer) was associated with increased risk of MACE (*P* = 0.006). After adjustments were made for LVEF and eGFR, the adjusted HR of each 1-SD higher log (D-dimer) was 6.980 (1.473–33.082). For male patients, the univariate cox regression analysis indicated that serum fibrinogen, log (D-dimer), log (NT-proBNP) and log [Lp(a)] were associated with increased risk of MACE (all *P* < 0.05). In adjusted Cox proportional hazards model, the adjusted HR of each 1-SD higher log (D-dimer) and log [Lp(a)] were 4.577 (1.802–11.624) and 2.176 (1.196–3.956), respectively. For female patients, the univariate cox regression analysis indicated that serum fibrinogen, log (D-dimer) and log (NT-proBNP) were associated with increased risk of MACE (all *P* < 0.05). In adjusted Cox proportional hazards model, the adjusted HR of each 1-SD higher fibrinogen, log (D-dimer) and log (NT-proBNP) were 1.453 (1.034–2.041), 7.265 (2.700–19.548), and 2.610 (1.422–4.792), respectively.

**Table 2:** Biomarkers and risk of major adverse cardiovascular event (MACE) after ACS.

	**Model 1**		**Model 2**	
**Biomarkers**	**HR (95% CI)**	***P*** **-value**	**HR (95% CI)**	***P*** **-value**
ACS patients				
Log(hs-CRP)	1.427 (1.018–2.000)	0.039	1.200 (0.838–1.717)	0.320
Fibrinogen	1.500 (1.187–1.895)	0.001	1.414 (1.120–1.777)	0.003
Log (D-dimer)	7.426 (3.931–14.028)	<0.001	6.028 (3.087–11.770)	<0.001
Log (NT-proBNP)	2.164 (1.568–2.987)	<0.001	2.000 (1.350–2.962)	0.001
Log [Lp(a)]	2.098 (1.332–3.304)	0.001	1.796 (1.149–2.806)	0.010
UA	1.001 (0.998–1.004)	0.508	0.999 (0.996–1.002)	0.620
Log (GGT)	1.470 (0.682–3.167)	0.326	1.283 (0.586–2.810)	0.534
**UAP patients**				
Log(hs-CRP)	1.180 (0.703–1.980)	0.530	1.126 (0.677–1.870)	0.648
Fibrinogen	1.429 (1.044–1.955)	0.026	1.376 (1.022–1.851)	0.035
Log (D-dimer)	5.426 (2.234–13.178)	<0.001	4.955 (2.051–11.971)	<0.001
Log (NT-proBNP)	4.128 (2.508–6.795)	<0.001	4.073 (2.382–6.967)	<0.001
Log (Lp [a])	1.617 (0.950–2.753)	0.077	1.604 (0.943–2.730)	0.082
UA	1.002 (0.999–1.006)	0.246	1.000 (0.997–1.004)	0.848
Log (GGT)	1.245 (0.476–3.261)	0.655	1.004 (0.376–2.684)	0.994
**NSTEMI patients**				
Log(hs-CRP)	1.989 (0.766–5.168)	0.158	1.587 (0.545–4.618)	0.397
Fibrinogen	3.053 (1.632–5.710)	<0.001	3.684 (1.768–7.679)	0.001
Log (D-dimer)	18.059 (4.173–78.156)	<0.001	21.265 (3.863–117.052)	<0.001
Log (NT-proBNP)	1.112 (0.340–2.377)	0.830	1.401 (0.353–2.589)	0.341
Log [Lp(a)]	5.770 (1.371–24.288)	0.017	4.833 (1.108–21.069)	0.036
UA	0.997 (0.989–1.006)	0.509	0.996 (0.987–1.005)	0.364
Log (GGT)				
STEMI patients	1.180 (0.154–9.047)	0.874	0.771 (0.093–6.387)	0.809
Log(hs-CRP)	1.589 (0.768–3.287)	0.212	1.529 (0.707–3.309)	0.281
Fibrinogen	1.079 (0.625–1.863)	0.785	1.059 (0.616–1.823)	0.835
Log (D-dimer)	7.180 (1.754–29.384)	0.006	6.980 (1.473–33.082)	0.014
Log (NT-proBNP)	1.709 (0.675–4.326)	0.258	1.546 (0.600–3.979)	0.367
Log (Lp [a])	1.418 (0.484–4.158)	0.525	1.476 (0.502–4.338)	0.479
UA	0.999 (0.994–1.005)	0.769	0.998 (0.992–1.004)	0.530
Log (GGT)	2.658 (0.475–14.866)	0.266	3.128 (0.544–17.982)	0.201
**Male patients**				
Log(hs-CRP)	1.589 (1.001–2.523)	0.050	1.310 (0.805–2.134)	0.277
Fibrinogen	1.471 (1.075–2.015)	0.016	1.360 (0.998–1.854)	0.052
Log (D-dimer)	6.451 (2.711–15.352)	<0.001	4.577 (1.802–11.624)	0.001
Log (NT-proBNP)	2.024 (1.304–3.143)	0.002	1.629 (0.968–2.739)	0.066
Log [Lp(a)]	2.357 (1.281–4.337)	0.006	2.176 (1.196–3.956)	0.011
UA	1.002 (0.998–1.005)	0.426	0.999 (0.995–1.003)	0.669
Log (GGT)				
Female patients	1.908 (0.653–5.577)	0.238	1.641 (0.560–4.808)	0.366
Log(hs-CRP)	1.271 (0.775–2.086)	0.342	1.107 (0.649–1.888)	0.708
Fibrinogen	1.521 (1.069–2.164)	0.020	1.453 (1.034–2.041)	0.031
Log (D-dimer)	8.826 (3.392–22.966)	<0.001	7.265 (2.700–19.548)	<0.001
Log (NT-proBNP)	2.362 (1.468–3.801)	<0.001	2.610 (1.422–4.792)	0.002
Log [Lp(a)]	1.301 (0.674–2.508)	0.433	1.408 (0.721–2.752)	0.316
UA	1.000 (0.996–1.005)	0.835	0.998 (0.993–1.003)	0.406
Log (GGT)	1.137 (0.377–3.430)	0.819	0.895 (0.285–2.807)	0.849

*hs-CRP, high-sensitivity C-reactive protein; NT-proBNP, N-terminal proB-type natriuretic peptide; Lp(a), lipoprotein(a); UA, uric acid; GGT, gamma-glutamyl transferase; UAP, unstable angina pectoris; NSTEMI, non-ST-segment elevation myocardial infarction; STEMI, ST-segment elevation myocardial infarction; HR, hazard ratio; CI, confidential intervals. Model1 was an unadjusted cox regression model. Model 2 was adjusted for LVEF and eGFR*.

ROC analysis showed that the optimal cutoff values of the biomarkers for predicting MACE were 3.26 g/L for fibrinogen (sensitivity: 62.96% and specificity: 53.70%), 0.87 ug/ml for D-dimer (sensitivity: 33.33% and specificity: 94.26%), 169 pg/ml for NT-proBNP (sensitivity: 70.37% and specificity:56.48%), and 77.9 mmol/l for Lp(a) (sensitivity: 43.21% and specificity: 72.96%). The area under the curve (AUC) was 0.591 (95% CI:0.521–0.660; *P* = 0.008) for fibrinogen, 0.647 (95% CI:0.575–0.719; *P* < 0.001) for D-dimer, 0.652 (95% CI:0.589–0.714; *P* < 0.001) for NT-proBNP, and 0.588 (95% CI:0.517–0.658; *P* = 0.011) for Lp(a). As shown in Table 3, when considered as a categorical variable, the adjusted HRs of higher fibrinogen, D-dimer, NT-proBNP and Lp(a) were 1.780 (1.131–2.800), 5.271 (3.216–8.640), 2.452 (1.466–4.102), and 1.942 (1.250–3.017), respectively.

**Table 3:** Elevated biomarkers and risk of MACE after ACS.

**Elevated biomarkers**	**HR (95% CI)**	**Sensitivity, %**	**Specificity, %**
Fibrinogen, ≥3.26 g/L	1.780 (1.131–2.800)	62.96	53.70
D-dimer, ≥0.87 ug/ml	5.271 (3.216–8.640)	33.33	94.26
NT-proBNP, ≥169 pg/ml	2.452 (1.466–4.102)	70.37	56.48
Lp(a), ≥77.9 mmol/L	1.942 (1.250–3.017)	43.21	72.96

*NT-proBNP, N-terminal proB-type natriuretic peptide; Lp(a), lipoprotein(a); HR, hazard ratio; CI, confidential intervals. Optimal cutpoints for fibrinogen, D-dimer, NT-proBNP, and Lp(a) were obtained from the receiver operating characteristics curves. MACE, major adverse cardiovascular event; ACS, acute coronary syndrome; HR, hazard ratio; CI, confidential intervals. The multivariable model included LVEF and eGFR*.

### Multiple biomarkers and clinical outcome

**Figure 2:**
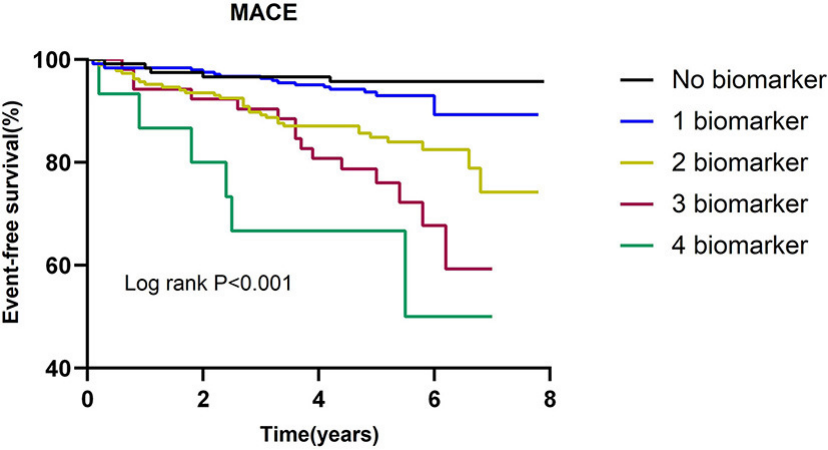
Kaplan-Meier survival curve for MACE according to number of elevated biomarkers.

The incidence of MACE in relation to the number of elevated biomarkers is shown in Table 4. A total of 15 patients had all 4 biomarkers elevated. Patients with a higher number of elevated biomarkers had an increased risk of MACE (*P for trend* < 0.001). The univariate cox regression analysis indicated that in comparison to the patients without any elevated biomarkers, the HR of those with 4 elevated biomarkers was 7.508 (2.602–21.662). After adjustment for LVEF and eGFR, the adjusted HR of patients with 4 elevated biomarkers was 6.008 (1.965–18.367). As shown in Figure 2, Kaplan-Meier survival analysis showed that the cumulative incidence of MACE increased with an increasing number of elevated biomarkers.

**Table 4:** Multivariable-adjusted HRs (95% CI) of MACE according to the number of elevated biomarkers among ACS patients.

**No. of biomarkers**	**Events**, ***n*** **(%)**	**Unadjusted HR (95% CI)**	***P***_trend_ **value**	**Adjusted HR (95% CI)**	***P***_trend_ **value**
0 (*n =* 121)	8 (6.6)	Reference	<0.001	Reference	<0.001
1 (*n =* 244)	18 (7.4)	1.111 (0.483–2.555)		1.124 (0.488–2.592)	
2 (*n =* 187)	32 (17.1)	2.655 (1.223–5.763)		2.498 (1.141–5.466)	
3 (*n =* 54)	17 (31.5)	5.162 (2.226–11.968)		4.746 (2.019–11.157)	
4 (*n =* 15)	6 (40.0)	7.508 (2.602–21.662)		6.008 (1.965–18.367)	

*MACE, major adverse cardiovascular event; ACS, acute coronary syndrome; HR, hazard ratio; CI, confidential intervals. The multivariable model included LVEF and eGFR*.

**Figure 3:**
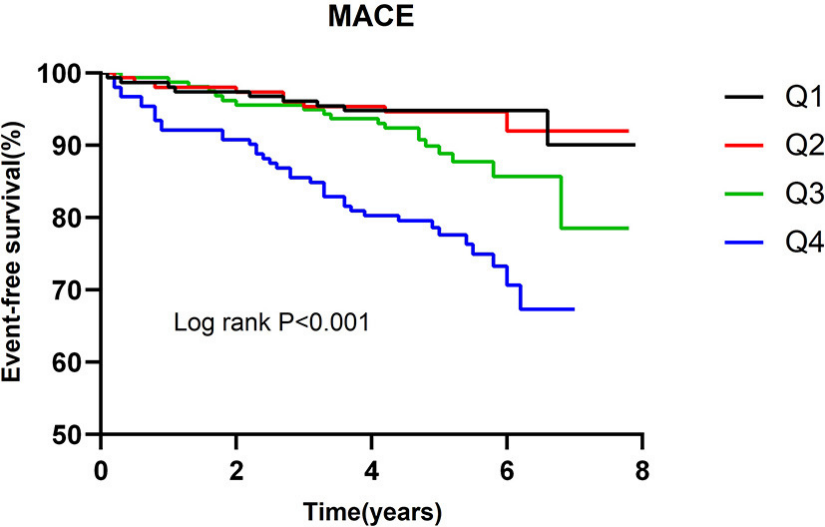
Kaplan-Meier survival curve for MACE across multimarker scores quartiles.

The incidence of MACE in relation to quartiles of the multimarker scores is presented in Table 5. Patients with a higher multimarker score had an increased risk of MACE (*P for trend* < 0.001). The univariate cox regression analysis indicated that the HR for MACE for quartile 4 of the multimarker score (relative to quartile 1) was 4.604 (2.304–9.198). In the multivariable analysis, the adjusted HR for MACE for quartile 4 of the multimarker score (relative to quartile 1) was 4.098 (2.003–8.384). As shown in Figure 3, Kaplan-Meier survival analysis showed that the cumulative incidence of MACE increased with higher quartiles of the multimarker score.

**Table 5:** Multivariable-adjusted HRs (95% CI) of MACE according to quartiles of multimarker scores among ACS patients.

**Multimarker score**	**Events**, ***n*** **(%)**	**Unadjusted HR (95% CI)**	***P***_trend_ **value**	**Adjusted HR (95% CI)**	***P***_trend_ **value**
Q1 (*n* = 155)	10 (6.5)	Reference	<0.001	Reference	<0.001
Q2 (*n =* 153)	11 (7.2)	1.128 (0.479–2.656)		1.106 (0.470–2.606)	
Q3 (*n =* 158)	19 (12.0)	1.920 (0.892–4.130)		1.863 (0.862–4.025)	
Q4 (*n =* 155)	41 (26.5)	4.604 (2.304–9.198)		4.098 (2.003–8.384)	

*MACE, major adverse cardiovascular event; ACS, acute coronary syndrome; HR, hazard ratio; CI, confidential intervals. The multimarker score was calculated as:[0.164x(Fibrinogen)] +[0.478x [log Lp(a)]] +[0.430x (log NT-proBNP)] +[1.530x (log D-dimer)]. The multivariable model included LVEF and eGFR*.

### Incremental prognostic value of biomarkers beyond that of established prognostic factors

The incremental prognostic values of the biomarkers for MACE are shown in Table 6. Adding log (D-dimer) or log [Lp(a)] to the basic model of established risk factors significantly improved the reclassification of MACE in terms of NRI (D-dimer: 47.3%, *P* < 0.001; Lp(a): 23.6%, *P* = 0.048) and IDI (D-dimer:6.2%, *P* < 0.001; Lp(a):1.3%, *P* = 0.012). Adding log (NT-proBNP) to the model of established prognostic factors improved the prediction of MACE (*P* = 0.034). Moreover, adding NT-proBNP also significantly improved the reclassification of MACE in terms of NRI (43.5%, *P* < 0.001) and IDI (2.2%, *P* < 0.001).

**Table 6:** Additional predictive value provided by candidate biomarker beyond that of the basic model.

**Models**	**C-index**	***P-value***	**NRI (95% CI)**	***P-value***	**IDI (95% CI)**	***P-value***
Basic model	0.592 (0.522–0.662)					
Basic model+ Fibrinogen	0.635 (0.567–0.702)	0.104	0.159 (−0.072–0.390)	0.181	0.027 (0.002–0.029)	0.027
Basic model+ Log (D-dimer)	0.657 (0.585–0.728)	0.058	0.473 (0.243–0.703)	<0.001	0.062 (0.037–0.087)	<0.001
Basic model+ Log (NT-proBNP)	0.651 (0.588–0.714)	0.034	0.435 (0.207–0.662)	<0.001	0.022 (0.010–0.035)	<0.001
Basic model+ Log [Lp(a)]	0.624 (0.557–0.690)	0.242	0.236 (0.004–0.467)	0.048	0.013 (0.003–0.023)	0.012
Basic model+ all 4 biomarkers	0.707 (0.638–0.776)	0.002	0.549 (0.323–0.776)	<0.001	0.086 (0.057–0.115)	<0.001
Basic model+ multimarker score	0.711 (0.643–0.778)	<0.001	0.549 (0.323–0.776)	<0.001	0.088 (0.058–0.118)	<0.001

*NT-proBNP, N-terminal proB-type natriuretic peptide; Lp(a), lipoprotein(a); NRI, net reclassification improvement; IDI, integrated discrimination improvement; CI, confidential intervals. The basic model included LVEF and eGFR*.

The C-statistic of the basic model of established risk factors for MACE was 0.592 (0.522–0.662) for MACE. Simultaneously adding these 4 additional biomarkers to the basic model significantly improved the prediction of MACE in terms of the C-statistic (from 0.592 to 0.707, *P* = 0.002), NRI (54.9%, *P* < 0.001), and IDI (8.6%, *P* < 0.001). The addition of the multimarker score to the basic model also improved the prognostic performance for predicting MACE in terms of the C-statistic (from 0.592 to 0.711 *P* < 0.001), NRI (54.9%, *P* < 0.001), and IDI (8.8%, *P* < 0.001).

## Discussion

In the present study, the prognostic utility of 7 circulating biomarkers for adverse cardiovascular events in patients with ACS without SMuRFs was investigated. The major findings were as follows. First, among the 7 biomarkers examined in the present study, higher fibrinogen, D-dimer, NT-proBNP and Lp(a) levels each were independently associated with increased risk of MACE. In contrast, hs-CRP, UA and GGT were not found to be useful predictive markers in the multivariate analyses. Only D-dimer, NT-proBNP and Lp(a) independently and significantly improved the reclassification of the risk for adverse cardiovascular events. Second, when all of the 4 associated biomarkers were incorporated together, there was a clear increase in the risk of MACE with an increasing number of elevated biomarkers. Patients with 4 elevated biomarkers had a 6.008-fold increased risk for MACE compared to those without any elevated biomarkers. The multimarker score combing these 4 markers was significantly associated with risk of MACE after adjusting for confounding covariables. Third, the addition of these 4 biomarkers or the multimarker score to the basic model significantly improved the risk prediction for MACE in patients with ACS without SMuRFs. These results indicate that fibrinogen, D-dimer, NT-proBNP and Lp(a) measurement may contribute to early identification of high-risk patients with ACS in the absence of traditional risk factors. Most importantly, these findings confirmed the superior predictive performance of the combination of multiple biomarkers for MACE in patients with ACS without SMuRFs.

Patients with ACS without SMuRFs represent heterogeneous class of patients, and these patients have worse long-term clinical prognosis than those with at least one SmuRF (5, 7). Recent evidence has demonstrated that patients with CAD without SMuRFs had similar rates of plaque progression as those with traditional risk factors (22). The reason for poor prognosis in these patients has not been clearly demonstrated. In the present study, there were no significant differences in the standard ischemic biomarkers including CK, CK-MB, and hs-TnT between the MACE group and the non-MACE group. It is likely that non-traditional risk factors such as inflammation, oxidative stress, activated coagulation, hemodynamic stress, and elevated levels of Lp(a) play a role in atherosclerosis progression (23–27). Hs-CRP, fibrinogen, D-dimer, NT-proBNP, Lp(a), UA, and GGT have been reported to be useful for stratifying patients by risk, independently of the patient's SMuRFs, and can improve risk prediction over that using only traditional risk factors (10, 15, 28–32). Together, these results suggest that rapidly available and reliable markers should be elucidated to help to identify patients with higher residual cardiovascular risk in the absence of traditional risk factors. To our knowledge, there is a lack of insight into the prognostic significance of biomarkers in patients with ACS without SMuRFs. Therefore, it is necessary to determine whether these biomarkers predict recurrent cardiovascular risk in these patients.

Our study indicated that fibrinogen, D-dimer, NT-proBNP, and Lp(a) were each significantly associated with MACE, either as continuous variables or as categorical variables. Moreover, the higher risk of MACE persisted after adjusting for potential confounding factors. Thus, each of these 4 biomarkers contributed statistically independent information toward risk stratification in patients without SMuRFs, and these results are consistent with the hypothesis that activated inflammation, activated coagulation, hemodynamic stress, and elevated levels of Lp(a) exacerbates cardiovascular risk. Therefore, the present study extended previous findings on the relation between biomarkers and prognosis to a novel population subtype of ACS. However, the predictive values of these 4 biomarkers varied significantly among patients with UAP, NSTEMI, or STEMI, which indicated that these biomarkers may not be applicable to all ACS subgroups simultaneously. Moreover, the predictive values of these 4 biomarkers also varied significantly between male and female patients. Only the prognostic value of D-dimer was applicable to male and female patients, as well as patients with UAP, NSTEMI, STEMI or the entire range of ACS patients. Furthermore, D-dimer had the strongest association with MACE. This suggests that activated coagulation may play a relatively more important role in the progression of atherosclerosis in patients without SMuRFs.

For patients with ACS without SMuRFs, each of the associated 4 biomarkers was suggested to be a viable piece of information for risk stratification. However, fibrinogen, D-dimer, NT-proBNP and Lp(a) each separately had only a modest predictive value for MACE, suggesting that only one biomarker cannot offer sufficient prognostic information. Moreover, when evaluated in the context of established risk factors, the incremental improvement upon adding fibrinogen, D-dimer or Lp(a) to the prognostic model was less marked in terms of the C-index. Only D-dimer, NT-proBNP, and Lp(a) significantly increased the NRI and IDI and provided additional information for predicting adverse cardiovascular events in patients with ACS without SMuRFs. As such, the relative prognostic value of D-dimer, NT-proBNP, and Lp(a) may be better than that of fibrinogen -in this specific class of ACS patients.

The incremental prognostic vaules of multiple biomarkers for cardiovascular events had been evaluated in patients with ACS (17–20). The present study was the first to focus on patients with ACS without SMuRFs. Fibrinogen, D-dimer, NT-proBNP and Lp(a) reflected distinct pathophysiological mechanism for poor prognosis in ACS, including inflammation, activated coagulation, cardiovascular stress and cholesterol metabolism. The results of this study demonstrated a clear relationship between an increasing number of elevated biomarkers and the risk of adverse cardiovascular events. By combing these 4 biomarkers into the multimarker score, a higher multimarker score was shown to be associated with a higher risk of MACE. Thus, the multimarker strategy that categorizes patients with ACS without SMuRFs based on the combination of these biomarkers seems to be of clinical utility. Furthermore, adding these 4 biomarkers to the basic model significantly improved the discrimination and reclassification of MACE. Of note, adding the multimarker score also significantly increased the C-index, NRI and IDI. More importantly, adding the 4 combined biomarkers to the basic model offered greater incremental value for risk prediction than adding any single one of the biomarkers, in terms of the C-index. This is consistent with the opinion that the multimarker strategy, which reflects different pathophysiological mechanisms, is relatively more informative and may provide complementary information (33). These results support the use of the multimarker strategy for risk prediction in patients with ACS without SMuRFs.

These findings have important clinical implications. These biomarkers are routinely utilized in clinical practice for patients with ACS. Simultaneous assessment of all 4 of these biomarkers could optimize risk stratification and enable clinicians to stratify cardiovascular risk more accurately. Our findings suggest that risk stratification based on the combination of fibrinogen, D-dimer, NT-proBNP and Lp(a) as well as clinical examination, is pragmatic for differentiating high-risk patients without SMuRFs in routine clinical practice. Considering its convenience and simplicity, simply utilizing number of elevated biomarkers may be a better choice. Current ACS guidelines do not specifically provide evidence-based management for patients without SMuRFs in the setting of secondary prevention. Recent studies have demonstrated the effectiveness of anti-inflammation, Lp(a)-lowering and NT-proBNP-guided therapies, as well as more intensive antithrombotic medications in patients at high risk for developing cardiovascular events (34–37). Therefore, these biomarkers may represent potential treatment targets for cardiovascular events in patients without traditional risk factors. In the present study, the optimal cutoff values of these biomarkers for predicting MACE were identified. Future studies are warranted to investigate whether reducing the levels of these biomarkers will be beneficial for patients without SMuRFs.

The present study had certain limitations which should be considered. First, it was a single-center retrospective study. Many biomarkers such as growth differentiation factor 15, lipoprotein-associated phospholipase, and clonal hematopoiesis of indeterminate potential were not included in the study; these should be studied further. Furthermore, patients with a history of cardiovascular disease were excluded from the current study to eliminate the influence of treatment on SMuRFs, which may limit the generalizability of these findings. Second, the analyses conducted in this study were based on a single measurement at admission. The multiple biomarkers were not further measured after treatment or discharge. Therefore, the predictive value of dynamic measurement of these biomarkers for cardiovascular events warrants further study. Third, due to the relatively small number of patients enrolled in the study and the relatively low incidence of cardiovascular events, the prognostic value of these established biomarkers for mortality were not analyzed further. Fourth, although a multimarker score to estimate the risk of MACE in patients without SMuRFs was constructed, no external validation of the multimarker score was not condcuted as part of the present study. Therefore, this finding requires further confirmation in a future study. Finally, we did not collect information about the compliance with guideline-directed medical management. The lack of information on the long-term status of medication use may have exaggerated the results of the study.

## Conclusion

Elevated fibrinogen, D-dimer, NT-proBNP, and Lp(a) levels were independent predictors of adverse cardiovascular events in patients with ACS without SMuRFs. Simultaneous assessment of these biomarkers significantly improved risk prediction for cardiovascular events. These findings suggest that strategies to improve risk stratification in patients with ACS without SMuRFs should incorporate biomarker data into risk algorithms.

## Data availability statement

The original contributions presented in the study are included in the article/supplementary material, further inquiries can be directed to the corresponding author/s.

## Ethics statement

The studies involving human participants were reviewed and approved by Tianjin Chest Hosptial. Written informed consent for participation was not required for this study in accordance with the national legislation and the institutional requirements.

## Author contributions

LW, H-lC, and J-xZ participated in the study design. LW, Y-cH, CW, J-cL, B-yZ, T-tL, C-wL, HY, L-bR, WQ, and W-yL participated in data collection. LW, X-mL, HY, and L-bR performed the statistical analysis. LW drafted the article. All authors read and approved the final manuscript.

## Conflict of interest

The authors declare that the research was conducted in the absence of any commercial or financial relationships that could be construed as a potential conflict of interest.

## Publisher's note

All claims expressed in this article are solely those of the authors and do not necessarily represent those of their affiliated organizations, or those of the publisher, the editors and the reviewers. Any product that may be evaluated in this article, or claim that may be made by its manufacturer, is not guaranteed or endorsed by the publisher.
